# Implementation of the PIERS on the Move mHealth Application From the Perspective of Community Health Workers and Nurses in Rural Mozambique

**DOI:** 10.3389/fgwh.2021.659582

**Published:** 2021-05-03

**Authors:** Helena Boene, Anifa Valá, Mai-Lei Woo Kinshella, Michelle La, Sumedha Sharma, Marianne Vidler, Laura A. Magee, Peter von Dadelszen, Esperança Sevene, Khátia Munguambe, Beth A. Payne, the CLIP Mozambique Working Group

**Affiliations:** ^1^Centro de Investigação em Saúde de Manhiça, Manhiça, Mozambique; ^2^Department of Obstetrics and Gynaecology, University of British Columbia, Vancouver, BC, Canada; ^3^Department of Women and Children's Health, King's College London, London, United Kingdom; ^4^Faculdade de Medicina, Universidade Eduardo Mondlane, Maputo, Mozambique

**Keywords:** PIERS on the Move, mHealth, community health workers, Mozambique, nurses

## Abstract

**Background:**mHealth is increasingly regarded as having the potential to support service delivery by health workers in low-resource settings. PIERS on the Move (POM) is a mobile health application developed to support community health workers identification and management of women at risk of adverse outcomes from pre-eclampsia. The objective of this study was to evaluate the impact of using POM in Mozambique on community health care workers' knowledge and self-efficacy related to caring for women with pre-eclampsia, and their perception of usefulness of the tool to inform implementation.

**Method:** An evaluation was conducted for health care workers in the Mozambique Community Level Intervention for Pre-eclampsia (CLIP) cluster randomized trial from 2014 to 2016 in Maputo and Gaza provinces (NCT01911494). A structured survey was designed using themes from the Technology Acceptance Model, which describes the likelihood of adopting the technology based on perceived usefulness and perceived ease of use. Surveys were conducted in Portuguese and translated verbatim to English for analysis. Preliminary analysis of open-ended responses was conducted to develop a coding framework for full qualitative analysis, which was completed using NVivo12 (QSR International, Melbourne, Australia).

**Results:** Overall, 118 community health workers (44 intervention; 74 control) and 55 nurses (23 intervention; 32 control) were surveyed regarding their experiences. Many community health workers found the POM app easy to use (80%; 35/44), useful in guiding their activities (68%; 30/44) and pregnant women received their counseling more seriously because of the POM app (75%; 33/44). Almost a third CHWs reported some challenges using the POM app (30%; 13/44), including battery depletion after a full day's activity. Community health workers reported increases in knowledge about pre-eclampsia and other pregnancy complications and increases in confidence, comfort and capacity to advise women on health conditions and deliver services. Nurses recognized the increased capacity of community health workers and were more confident in their clinical and technological skills to identify women at risk of obstetric complications.

**Conclusions:** Many of the community health workers reported that POM improved knowledge, self-efficacy and strengthened relationships with the communities they serve and local nurses. This helped to strengthen the link between community and health facility. However, findings highlight the need to consider program and systematic challenges to implementation.

## Introduction

Alongside the rapidly increasing availability and use of mobile phones in sub-Saharan Africa and other low- and middle-income countries (LMIC) has been a rise of mHealth - where mobile phones and tablets are used to strengthen health services in resource-limited settings (1, 2). mHealth applications developed for use by frontline health workers have demonstrated ability to improve quality of clinical data collection, adherence to treatment algorithms, streamline supervision and reporting and to maintain health worker motivation and skill level (3). In addition, for maternal health care delivery specifically, mHealth interventions can improve health decision-making of community level providers, such as community health workers (CHWs), to facilitate linkages to higher-level obstetric care (4).

Mozambique has a long history of CHWs; however, disorganized integration of CHWs in the health services structure has been a major limitation for the reach of program. Deterioration of the CHW program in Mozambique was exacerbated by the civil war (1976–1992), which resulted in a lack of funding, training and professional development opportunities, appropriate supervision and technical support and the destruction of hundreds of health facilities where CHWs previously worked (5, 6). The revitalized CHW program was launched in 2010 and expanded traditional CHW roles to include home visits and Integrated Community Case Management (iCCM) of malaria, pneumonia and diarrhea, the three deadliest illnesses for children under five in sub-Saharan Africa (5). While the WHO- and UNICEF-supported iCCM program included training and supplies, these were focused on child health and no similar program existed for maternal health conditions (5). CHW roles in pregnancy were limited to basic maternal health counseling, verification of antenatal care (ANC) attendance and promotion of hospital-based delivery (5, 7). While CHWs were expected to recognize pregnancy danger signs, they did not receive training or were not provided equipment to confirm diagnoses (5). The CHW training manual included nothing related to pre-eclampsia and eclampsia (7). Providing treatment in pregnancy was seen as outside the scope of CHW responsibilities; instead, their roles were to advise women to go to nearest local health facility when needed (5).

The Community Level Interventions for Pre-eclampsia (CLIP) trial in Mozambique used the PIERS on the Move (POM) mHealth application to support CHW capacity to identify, triage and provide initial management for pregnant women at risk of adverse outcomes related to pre-eclampsia (8). Complicating 10.9% of pregnancies in southern Mozambique (9), pregnancy hypertension is the third leading cause of maternal mortality in Mozambique (10–12). A study with CHW supervisors, district medical officers and gynecologists-obstetricians in Mozambique highlighted the importance of early identification of women with pregnancy hypertension and management at the community-level before referral (7). However, while CHW supervisors, district medical officers and gynecologists-obstetricians supported task-sharing to CHWs, they did not think it would be possible due to limited clinical knowledge, skills and training (7). The CLIP trial aimed to test the hypothesis that CHWs could take on additional clinical tasks to improve timely care of pregnancy hypertension. Unfortunately, the CLIP intervention including the POM app was not found to be effective in reducing maternal and/or neonatal death or major morbidity (AOR 1.31, 95% CI: 0.70–2.48) (8). Understanding how this complex health intervention worked within the health system setting is important in interpreting and learning from this effectiveness evaluation.

Previous research has demonstrated that the implementation of maternal and neonatal mHealth interventions were strengthened by ease of use, flexibility of use, adaptability and development within the local context (1). However, despite increasing uptake of mHealth apps globally, there is limited literature on the evaluation of applications for clinical decision support in pregnancy (13). Additional themes for evaluation of clinical decision support mHealth apps include acceptability and satisfaction, portability, versatility of smartphones to engage in multiple functions and user involvement in development and evaluation (13).

The objective of this study was to evaluate the impact of using POM in Mozambique on CHWs knowledge and self-efficacy related to caring for women with pre-eclampsia, and their perception of usefulness of the tool. We assessed, from the health care worker's perspective, the usability and acceptability of the POM application. Additionally, we aimed to understand the impact of this mHealth app on empowerment, self-efficacy, confidence and motivation, as well as how the CLIP intervention impacted their role within the broader health system.

## Methods

### Study Design and Area

This mixed-methods evaluation was designed for health care workers involved in the Mozambique Community Level Intervention for Pre-eclampsia (CLIP) cluster randomized controlled trial (NCT01911494) (8). The CLIP Mozambique trial was conducted from 2014 to 2016 in Maputo and Gaza provinces in southern Mozambique (8). The intervention was a complex package that consisted of community engagement activities and CHW visits with pregnant women in their homes or primary health centers, visits focused on the detection and management of pregnancy hypertension. The CLIP Mozambique trial aimed to reduce maternal morbidity and mortality through building capacity of nurses, supervisors and community health workers to better identify and triage women at risk using the PIERS On the Move (POM) mobile application (8, 14, 15). The POM app is a decision aid and was used to guide CHWs through a standard clinical assessment, to identify pregnancy hypertension and assess risk of complications using blood pressure, proteinuria and symptom evaluation. For the CLIP trial CHWs were trained to take on additional clinical skills, specifically: measuring blood pressure, testing urine for protein and measuring blood oxygen saturation through a pulse oximeter connected to their mobile device (14). More detailed information of the CLIP trial is described elsewhere (8).

The CLIP trial was conducted in Maputo and Gaza provinces. Provinces in Mozambique are divided into districts, administrative posts (AP), localities and neighborhoods. The APs in the study area (Maluana and Maciana, Calanga and Ilha Josina Machel, Três de Fevereiro, Magude, Messano, Chaimite, Xilembene, Chissano, Mazivila, Chongoene, Chicumbane and Malehice) are largely impoverished rural areas where the predominant occupations are agriculture, livestock rearing, informal trading, migrant labor (mainly to the capital city of Maputo and South Africa), handicrafts, and work in private sugar and rice farms. Residents of these APs are mostly of the Changana ethnic group and speak the respective dialect, *Xichangana*. In the region, there are 38 primary health centers staffed by nurses/midwives that offer reproductive and child health services, including deliveries for uncomplicated births (16). The region has a maternal mortality ratio of 204.6 deaths per 100,000 live births (16).

### Data Collection and Analysis

Data was collected using a structured survey that was designed using themes from the Technology Acceptance Model, which describes the likelihood of adopting technology based on perceived usefulness and perceived ease of use (17). The survey evaluated CHW and nurse confidence regarding their knowledge and skills on the management of pregnant women of CHWs and nurses involved in maternal and child care at the primary health center using 35 questions ranked on a five-point Likert scale. Additionally, CHWs who used the POM app as part of the CLIP intervention were asked 10 open-ended questions on their perspectives and experiences using the app, including the benefits, changes in behavior, any problems and recommended changes

The survey was administered between October and November 2017. Nurses and CHWs in both intervention and control clusters were surveyed, though CHWs from control clusters were not asked the open-ended questions because they did not use POM. Research assistants reached out to CHWs and nurses via phone. During recruitment, participants were briefed on the purpose and procedures of the study then asked if they wanted to participate; there were no refusals to participate.

Data collection was led by a social scientist from *Centro de Investigação em Saúde da Manhiça* (CISM) in Mozambique and assisted by three local research assistants. These researchers were selected due to their familiarity with the socio-cultural context, the research topic and their qualitative and quantitative data collection expertise. Team members were fluent in *Portuguese* and *Xichangana*, and had no prior personal relationship with the participants. Research assistants administered the survey questionnaire in person at the health facilities where the CHWs and nurses worked or in their homes. The survey was administered in *Portuguese* or *Xichangana*, depending on the preference of the participant, and took an average of 20 min.

**Figure 1:**
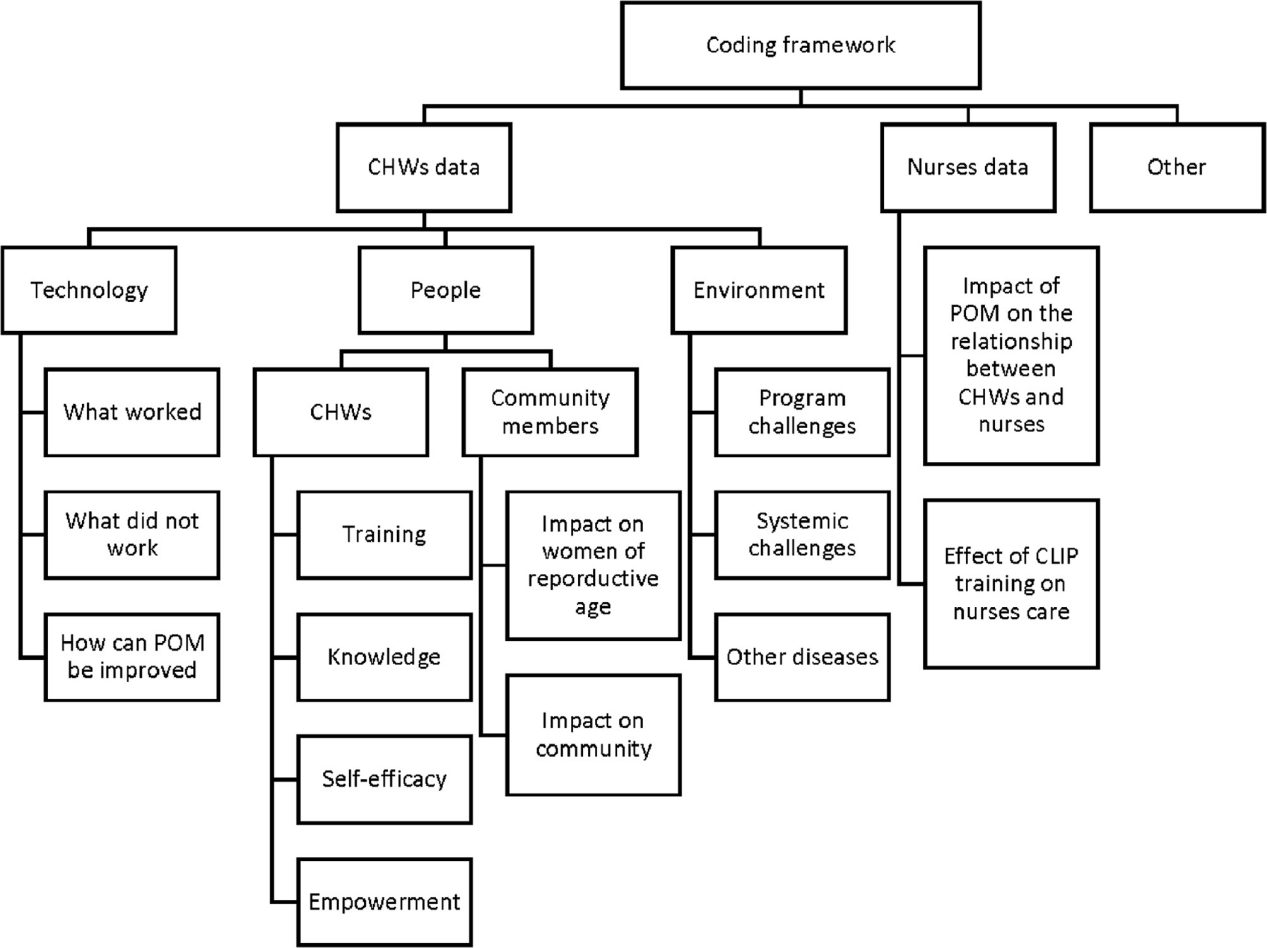
Qualitative coding framework.

The data were collected through electronic forms implemented in Open Data Kit (ODK) (an app for android based tablets). The survey responses were recorded directly in study tablets in Portuguese, which were then translated to English for analysis. Demographics, knowledge and self-efficacy data were exported to Stata13 (StataCorp, College Station, United States) for descriptive statistical analysis (absolute and relative frequencies, medians, and interquartile ranges; see Table 1 and Supplementary Tables 2–4). Qualitative analysis was undertaken for open-ended responses. Qualitative analysis was conducted collaboratively by Mozambican and Canadian social scientists (HB, AV, MKW, MV, BP) using NVivo12 (QSR International, Melbourne, Australia). Perceived usefulness and ease of use within the Technology Acceptance Model was incorporated into the coding framework as CHW's descriptions of the POM app on “what worked,” “what did not work,” and “how POM can be improved.” The coding framework also incorporated emergent themes such as “self-efficacy,” “empowerment,” “impact of POM on the relationship between CHWs and nurses” (see Figure 1). Thematic analysis was then performed to identify additional patterns in the qualitative data (18, 19).

**Table 1:** Participant's characteristics.

	**CHWs**			**Nurses**		
	**Intervention (*n* = 44)**	**Control (*n* = 74)**	**Total CHWs (*n* = 118)**	**Intervention (*n* = 23)**	**Control (*n* = 32)**	**Total nurses (*n* = 55)**
**Age (years)**						
Median (IQR)	39 (30, 48)	43 (36, 52)		29 (28, 42)	30 (26, 32)	
Range	22–72	24–68	22–72	22–58	23–57	22–58
**Gender**						
Male	12 (27.3%)	19 (25.7%)	31 (26.3%)	3 (13.0%)	0 (0.0%)	3 (5.5%)
Female	32 (72.7%)	55 (74.3%)	87 (73.7%)	20 (87.0%)	32 (100.0%)	52 (94.5%)
**Highest level education attained**						
Some primary level	6 (13.6%)	16 (21.6%)	22 (18.6%)	0 (0.0%)	0 (0.0%)	0 (0.0%)
Primary level completed	16 (36.4%)	24 (32.4%)	40 (33.9%)	1 (4.3%)	1 (3.1%)	2 (3.6%)
Incomplete secondary level	9 (20.5%)	5 (6.8%)	14 (11.9%)	4 (17.4%)	3 (9.4%)	7 (12.7%)
Secondary level completed	1 (2.3%)	2 (2.5%)	3 (2.5%)	17 (73.9%)	28 (87.5%)	45 (81.8%)
Higher degree	0 (0.0%)	0 (0.0%)	0 (0.0%)	1 (4.3%)	0 (0.0%)	1 (1.8%)
Missing information	12 (27.3%)	27 (36.5%)	39 (33.1%)	0 (0.0%)	0 (0.0%)	0 (0.0%)
**Marital status**						
Single	13 (29.5%)	20 (27.0%)	33 (28.0%)	0 (0.0%)	17 (53.1%)	17 (30.9%)
Married	24 (54.5%)	42 (56.8%)	66 (55.9%)	6 (26.1%)	15 (46.9%)	21 (38.2%)
Divorced	2 (4.5%)	2 (2.7%)	4 (3.4%)	17 (73.9%)	0 (0.0%)	17 (30.9%)
Widowed	5 (11.4%)	10 (13.5%)	15 (12.7%)	0 (0.0%)	0 (0.0%)	0 (0.0%)
**Years of experiences as a CHW/nurse**						
Median (IQR)	5 (3, 8)	5 (3, 6)		8 (4, 12)	12 (8, 14)	
Range	2–33	1–38	1–38	1–37	1–33	1–37

### Ethical Considerations

Ethical approval for the study was granted by the CISM Institutional Review Board in Mozambique (CIBS- CISM/08/2013), as well as by the UBC C&W Research Ethics Board in Canada (H12-00132). Each participant provided informed consent form before data collection.

## Results

### Participant's Characteristics

Our study surveyed 118 CHWs and 55 nurses. The median age of all participants was 42 years, CHWs tended to be older than nurses, who had a median age of 29 years. A majority of healthcare workers interviewed were women (74% of CHWs; 95% of nurses). Among CHWs, 34% completed primary school as the highest education level attained and. Both CHWs and nurses had a median of 5 years work experience. There were 44 CHWs from intervention clusters and 74 CHWs from control clusters. There were also 22 nurses from intervention clusters and the 32 nurses from control clusters. Demographics did not vary for CHWs or nurses between intervention and control (Table 1).

### Nurses' Knowledge, Self-Efficacy, and Relationships

Nurses surveyed from both intervention and control clusters were confident in their knowledge and skill to care for pregnant women in their communities (see Supplementary Table 2). All nurses agreed or strongly agreed that they were confident in recognizing pregnancy complications and signs of labor, giving health advice and counseling women to seek medical attention. All but one nurse agreed or strongly agreed that they could recognize symptoms of pre-eclampsia and all nurses reported that they could recognize eclampsia and could accurately check blood pressure. Similarly, nurses from both intervention and control clusters felt that women and families trusted the care they provided (96% agreed/strongly agreed in intervention cluster vs. 100% in control clusters) and hospital doctors respected their skills in identifying maternal complications (96% agreed/strongly agreed in intervention cluster vs. 100% in control clusters).

In comparison to 41% of nurses in control clusters, 87% nurses in intervention clusters agreed or strongly agreed they felt comfortable working with the CHWs in their communities. Similarly, more nurses from intervention clusters compared to nurses from control clusters trusted CHW's assistance (83% agree/strongly agree in intervention vs. 41% in control), particularly to support women to attend antenatal care earlier (91 vs. 41%), identify women with obstetric emergencies (87 vs. 41%) and early referral of pregnant women at risk of adverse outcomes (91 vs. 41%).

### CHWs' Knowledge, Self-Efficacy, and Relationships

Most CHWs reported confidence in their knowledge and skill to care for pregnant women in their communities (see Supplementary Table 3). This included confidence in providing basic information about pregnancy (100% agree/strongly agree in intervention cluster vs. 89% in control clusters), recognizing pregnancy-related complications (100 vs. 91%), and recognizing the signs and symptoms of labor (98 vs. 88%).

In comparison to 20% of CHWs in control clusters, 100% of CHWs in intervention clusters agreed or strongly agreed they could confidently recognize danger signs and symptoms related to pre-eclampsia. Similarly, more CHWs from intervention clusters said they could confidently recognize danger signs and symptoms related to eclampsia (98% agree/strongly agree in intervention cluster vs. 19% in control clusters) and could accurately measure blood pressure in pregnant women (100 vs. 60%). More CHWs from intervention clusters could administer injectable medicines in mothers and children (84 vs. 51%) and specifically administer intramuscular injections to pregnant women at risk of seizures (98 vs. 19%).

Most of the CHWs, in both intervention (98%) and control clusters (93%) agreed or strongly agreed that women and their families sought their advice for maternal health and trusted their ability to care for pregnant women in their communities. Most CHWs also felt that nurses respected their judgment in identifying pregnant women in need of care (98% agree/strongly agree in intervention clusters vs. 92% in control clusters) and that doctors and nurses valued their role as a CHW (100 vs. 95%). Most CHWs felt supported by supervisors to identify pregnant women in their communities (98 vs. 92%).

### CHWs' Experiences of Using POM App

#### Usability of the POM App

Four out of five CHWs from the intervention clusters (80%) agreed or strongly agreed that the POM app was easy to use and over two-thirds (68%) felt it was useful in advising women about pregnancy complications (see Supplementary Table 4). This was also reflected in the qualitative data where CHWs elaborated that the POM app was useful in guiding their activities and contained important information for communities regarding maternal health. Additionally, electronic data entry was reported by CHWs to increase the usability of the device.

“It saved all the women's information properly. I believe that if we had needed to write this all out on paper we'd have lost a lot of information, but the POM, from my point of view, is a secure instrument.” *Male CHW from Gaza province*

All CHWs that used POM agreed or strongly agreed that images were useful in helping them counsel women and that pictograms accurately represented clinical symptoms. CHWs shared that the images were well-received, particularly within the context of low literacy rates.

“For example, I'll talk about the vomiting, rather than talking about everything; the women are able to see that the woman is vomiting because it's all clear in the images.” *Female CHW from Maputo province*

“It was very easy to interpret because the images were clear. Even for someone who couldn't read, they were able to interpret it easily.” *Female CHW from Gaza province*

Almost all (98%) agreed or strongly agreed they have been effectively trained to use the POM app, the training provided them the necessary skills to care for pregnant women, and refresher trainings helped them overcome obstacles. Additionally, almost all CHWs reported that their supervisors actively supported their use of the POM app (93% agree/strongly agree).

#### Challenges Using the POM App

Almost a third of CHWs (30%) reported they had problems completing visits using the POM app (see Supplementary Table 4). These problems included poor connection with the pulse oximeter attachment and battery life during a full day of community visits.

“The oximeter sometimes did not count well. It was a connection problem.” *Male CHW from Maputo province*

“The POM's battery charge wasn't always sufficient. Some days when we had several visits, the POM's battery would die before we finished the visits so I had to stop to go home and charge it and continue the next day.” *Female CHW from Gaza province*

“It (the tablet) wasn't always charged because there is no power here at my workplace, and on days when it rained and I had no power, it didn't work.” *Female CHW from Maputo province*

However, most CHWs felt supported to solve problems encountered during their use of the POM app (91% agree/strongly agree).

#### Building Maternal Health Knowledge and Clinical Skills

All CHWs reported that they felt good about using the POM app (see Supplementary Table 4). CHWs elaborated that they learned about pre-eclampsia as a serious condition for pregnant women, which they were previously unaware of, and learned new clinical skills.

“I thought that when a pregnant woman's ankles got swollen, it was because she was going to have twins, and when they had pre-eclampsia, I thought it was moon sickness that she hadn't managed to treat when she was a child, but now I know that it's a sickness.” *Female CHW from Gaza province*

“I learned a lot of things that I didn't know. I didn't know how to measure blood pressure, I didn't know how to administer an injection in a pregnant woman, but in the study, I learned how to do it. I'm grateful for the good experience.” *Female CHW from Gaza province*

“…I saw that I could do the same thing without the help of a nurse. I had the information and with instruction from the POM, I administered it (MgSO_4_).” *Male CHW from Gaza province*

A number of CHWs spoke of increased confidence and capacity to advise pregnant women on health conditions. CHWs felt empowered by learning about pregnancy complications, their management and the impact of early intervention.

“I was more knowledgeable and smart enough to be able to do what I never imagined I could do, and today I feel able to face several difficulties with my head held high.” *Female CHW from Gaza province*

“I felt more important knowing that I'm caring for lives and that I'm saving a lot of people.” *Female CHW from Gaza province*

#### Strengthening Relationships With Communities

Almost all CHWs felt that pregnant women reacted positively to the POM app (93% agree/strongly agree) and a majority of CHWs felt that women took their counseling more seriously because of the POM app (75% agree/strongly agree) (see Supplementary Table 4). CHWs described how women previously avoided their health visits. With use of POM, community members valued the added maternal health screenings, which was seen to help with earlier identification of health concerns and more precise referrals to health facilities.

“I was respected, I valued my work, although there were women who at first preferred to flee their homes because they did not want our visits but after realizing that they would receive important care, they started to give value.” *Female CHW from Gaza province*

“I liked it a lot because I learned a lot with POM and it was valued more in the community. The mothers did not spend as much time in the hospital and they could come by and say thank you.” *Female CHW from Maputo province*

“A lot changed because a lot of people knew right away that she was sick and referred her quickly to the health unit.” *Female CHW from Maputo province*

## Discussion

Our research found that both the CHWs who used POM and those who did not use POM were confident in their knowledge to care for pregnant women in their communities. However, while overall self-reported knowledge and self-efficacy did not differ between groups, CHWs who used the POM app valued the training to further their skills and expand the clinical maternal health services they could provide, particularly the detection and management of pregnancy hypertension. CHWs from intervention clusters reported that POM was easy to use, contained useful information in the care of pregnant women and particularly appreciated guidance on clinical skills. Images improved communication of complex maternal health conditions. Skills to provide accurate clinical information were reported to increase respect from both community members and nurses. POM consequently served as a means of strengthening the connection of CHWs to the health system and their relationship with nurses. A similar finding reported by a systematic review showed that the use of mHealth changed how health workers interacted and brought the possibility of having more connections between colleagues that improved the coordination and the quality of care (20).

This study demonstrated that CHWs felt confident using a mobile health tool while caring for pregnant women. Previously, most CHWs in these communities could not measure blood pressure or proteinuria (90%) and provide treatment for initial management of pre-eclampsia in the community (86% not confident in providing oral antihypertensive and 95% for injections in pregnancy) (7). Additionally, only two out of five CHWs (41%) were able to describe the signs and symptoms of pregnancy hypertension prior to the study (7). Though aware of pregnancy complications, CHWs were previously limited in their knowledge and skill around pre-eclampsia and eclampsia (7). Frontline workers are often overburdened with community health responsibilities yet lack the support in terms of training, supplies, equipment and supportive supervision (21). This challenge compromises performance and damages respect from the community and health system (21). The POM app facilitated expansion of clinical skills supported by ongoing CLIP team supervision as well as warning alerts built into the application.

Demonstrated capacity to perform new clinical tasks increased CHW's self-esteem as well as respect from the community and nurses. Comments by CHWs who used POM shared that they felt empowered from being able to diagnose and manage a potentially life-threatening situation. Increased respect in the communities was also found with the CRADLE Vital Signs Alert, a blood pressure device implemented in low-resource settings (22). The CRADLE device was used in the CLIP trial, though the acceptability and feasibility were evaluated separately (22). A review also found mobile applications for clinical decision support in pregnancy increased health worker confidence and strengthened positive relationships and trust in the health provider (13). While it was not surprising that knowledge and confidence did not differ between nurses in the intervention and control clusters since nurses did not use POM, it is meaningful that more nurses in intervention clusters reported feeling comfortable working with the CHWs while they were using POM and trusted their knowledge and skill to care for pregnant women. It indicates that the relationship between nurses and CHWs was positively impacted by introduction of the POM app. A mHealth study in Burkina Faso that paired CHWs with professionally-trained health care workers also found enhanced communication and collaboration to provide better maternal care (23).

There is potential with appropriate training, supervision, and technologies that community health workers can play a more substantial role in maternal health through clinical identification and emergency management of pregnancy complications. In addition to the support of POM in guiding CHWs on a broader scope of clinical services, strengthening self-esteem and health system linkages also resulted from implementing the application. Critical to the expanded success of CHWs providing maternal health care will be ensuring they are able to reach women most in need. Failure of the CLIP intervention, including the POM app to improve maternal and neonatal health outcomes may have resulted from limitations in the reach of the intervention. In Mozambique, 60.3% of women recruited to the trial saw a CHW using the POM app at least once during pregnancy but only 11.0% received eight visits or more. In secondary analysis of the trial data, it was found that women who received eight CHW visits did have better maternal and neonatal outcomes (OR 0.79; 95% CI 0.63–0.99) (24). Any expansion of CHW scope must therefore also include increases in health workforce to ensure adequate coverage and should address limitations of technology, such as device battery life.

A limitation of the study was that we were not able to contact all CHWs and nurses in study areas. Some CHWs and nurses were not reached because their numbers changed in the course of the study. We were unable to reach seventeen of the 135 CHWs in the study area. The use of Likert scales to assess self-efficacy and confidence does not allow in-depth analysis. The study could have been strengthened with a clinical skill and knowledge assessment to ascertain differences in skillset, rather than simply confidence. The questionnaire design was not specific to the different roles related to use of the POM app and the responses may reflect confidence in different sets of skills. For CHWs in intervention clusters, this may reflect self-efficacy using POM while for CHWs in control clusters, it may have reflected self-efficacy in other tasks. This highlights the importance of the qualitative data in our study. Most of the control CHWs were from a cluster where there was another intervention with CHWs. This may have led to some of the higher efficacy responses.

Additionally, when necessary for CHWs that had low literacy, research assistants asked survey questions aloud in the local language, *Xichangana*, and filled out the questionnaire on participants' behalf. The interviewers made it very clear that they would fill out the form with the answers given without correction or judgment. For the nurses, it was difficult to have time to fill out the forms due to the dynamics of their work and because of that, some of the answers may have been rushed.

Furthermore, the current study evaluated health worker perceptions of implementing the POM app and their self-reported reflections on how it was received in the communities they worked in. A limitation of the study is that perceptions of pregnant women receiving care and other community members were not elicited or confronted with CHW's views. This is an area of potential future research.

Despite these limitations, this study has many strengths. To complement the surveys responses, CHWs were asked open-ended questions to elaborate about their experiences. Local researchers familiar with the study area and socio-cultural context conducted data collection and analysis. Little literature is available regarding the interaction of community health worker and maternal and child health nurses in relation to maternal health care in Mozambique.

## Conclusion

Community health workers reported that POM improved knowledge, self-efficacy and strengthened relationships with the communities they serve. Community health workers especially appreciated the development of clinical skills, which bolstered community prestige and helped them feel more part of the health system. Concurrently, nurses providing direct supervision to CHWs reported an increase in confidence in CHW skills and knowledge and increased willingness to support their decisions in maternal health care. This study demonstrates that it is possible to build clinical and technological capacity of community health workers. However, findings also highlight the need to consider program and systematic challenges to implementation.

## Data Availability Statement

De-identified raw data supporting the conclusions of this article will be made available by the authors, without undue reservation.

## Ethics Statement

The studies involving human participants were reviewed and approved by CISM Institutional Review Board in Mozambique (CIBS-CISM/08/2013) and the UBC C&W Research Ethics Board in Canada (H12-00132). The patients/participants provided their written informed consent to participate in this study.

## Author Contributions

PD, ES, KM, and LM conceptualized the trial and components of the intervention. BP, ES, and KM conceptualized the health worker evaluation. HB, AV, ML, SS, BP, and MV supported the implementation of the trial and health worker evaluation. HB, AV, M-LK, and BP performed the analysis and wrote the first draft of the manuscript. All authors provided feedback and review of the manuscript.

## Conflict of Interest

The authors declare that the research was conducted in the absence of any commercial or financial relationships that could be construed as a potential conflict of interest.
