# Supplementary material for: Implementation of the PIERS on the Move mHealth Application From the Perspective of Community Health Workers and Nurses in Rural Mozambique
Source: Front Glob Womens Health. 2021 May 3;2:659582. doi: 10.3389/fgwh.2021.659582 (PMC8593977; doi:10.3389/fgwh.2021.659582)
Supplement: Supplementary file 1 [file Table_1.DOCX]

**Supplementary tables**

**Table S1 CLIP Mozambique Working Group**

| **CLIP Mozambique Working Group** | |
| --- | --- |
| **First and middle names** | **Last names** |
| Felizarda | Amose |
| Ana Ilda | Biz |
| Rogério | Chiaú |
| Silvestre | Cutana |
| Paulo | Filimone |
| Marta | Macamo |
| Sónia | Maculuve |
| Ernesto | Mandlate |
| Analisa | Matavele |
| Sibone | Mocumbi |
| Dulce | Mulungo |
| Zefanias | Nhamirre |
| Ariel | Nhancolo |
| Cláudio | Nkumbula |
| Vivalde | Nobela |
| Rosa | Pires |
| Faustino | Vilanculo |
| Rahat N | Qureshi |
| Sana | Sheikh |
| Zahra | Hoodbhoy |
| Imran | Ahmed |
| Amjad | Hussain |
| Javed | Memon |
| Farrukh | Raza |
| Mrutunjaya B | Bellad |
| Shivaprasad S | Goudar |
| Ashalata A | Mallapur |
| Shashidhar G | Bannale |
| Umesh S | Charantimath |
| Keval S | Chougala |
| Richard J | Derman |
| Vaibhav B | Dhamanekar |
| Narayan V | Hoonungar |
| Anjali M | Joshi |
| Namdev A | Kamble |
| Chandrasekhar | Karadiguddi |
| Geetanjali M | Katageri |
| Avinash J | Kavi |
| Gudadayya S | Kengapur |
| Bhalachandra S | Kodkany |
| Uday S | Kudachi |
| Sphoorthi S | Mastiholi |
| Geetanjali I | Mungarwadi |
| Umesh Y | Ramadurg |
| Amit P | Revankar |
| Olalekan O | Adetoro |
| John O | Sotunsa |
| Sharla K | Drebit |
| Chirag | Kariya |
| Mansun | Lui |
| Diane | Sawchuck |
| Ugochi V | Ukah |
| Mai-Lei Woo | Kinshella |
| Shafik | Dharamsi |
| Guy A | Dumont |
| Tabassum | Firoz |
| Ana Pilar | Betrán |
| Susheela M | Engelbrecht |
| Veronique | Filippi |
| William A | Grobman |
| Marian | Knight |
| Ana | Langer |
| Simon A | Lewin |
| Gwyneth | Lewis |
| Craig | Mitton |
| Nadine | Schuurman |
| James G | Thornton |
| France | Donnay |
| Kelly | Pickerill |
| **CLIP Trial Working Group** | |
| Esperança Sevene, Eusébio Macete, Khátia Munguambe, Charfudin Sacoor, Anifa Vala, Helena Boene, Felizarda Amose, Rosa Pires, Zefanias Nhamirre, Marta Macamo, Rogério Chiaú, Analisa Matavele, Faustino Vilanculo, Ariel Nhancolo, Silvestre Cutana, Ernesto Mandlate, Salésio Macuacua, Quinhas Fernandes, Rosa Marlene Cuco, Cassimo Bique, Sibone Mocumbi, Emília Gonçálves, Sónia Maculuve, Ana Ilda Biz, Dulce Mulungo, Orvalho Augusto, Paulo Filimone, Vivalde Nobela, Corsino Tchavana, Cláudio Nkumbula  Jeffrey Bone, Dustin T Dunsmuir, Sharla K Drebit, Chirag Kariya, Mai-Lei Woo Kinshella, Tang Lee, Jing Li, Mansun Lui, Beth A Payne, Kelly Pickerill, Diane Sawchuck, Sumedha Sharma, Domena K. Tu, Marianne Vidler, Ugochi V Ukah, Laura A Magee, Peter von Dadelszen | |
| **CLIP Trial Adjudication Committee** | |
| Nafissa Osman, Cassimo Bique, Natercia Fernandes, Betuel Sigauque Raquel Gonzalez | |
| **CLIP Trial Steering Committee** | |
| J Mark Ansermino, Ana Pilar Betrán, Richard Derman, Shafik Dharamsi, France Donnay, Sharla Drebit, Guy Dumont, Susheela M. Engelbrecht, Veronique Fillipi, Tabassum Firoz, William Grobman, Marian Knight, Ana Langer, Simon Lewin, Gwyneth Lewis, Craig Mitton, Nadine Schuurman, Andrew H Shennan, Joel Singer, Jim Thornton, Hubert Wong | |
| **CLIP Trial Executive Committee** | |
| Olalekan Adetoro, Mrutunjaya M Bellad, Zulfiqar Bhutta, Peter von Dadelszen, Shivaprasad S Goudar, Jerker Liljestrand, Laura A Magee, Ashalata Mallapur, Khátia Munguambe, Beth Payne, Rahat Qureshi, Charfudin Sacoor, Esperança Sevene, Sumedha Sharma, John Obafemi Sotunsa, Marianne Vidler | |
| **CLIP Data Safety and Monitoring Board (DSMB**) | |
| Romano Nkumbwa Byaruhanga, Brian Darlow, Eileen Hutton, Mario Merialdi, Lehana Thabane | |

**Table S2. Nurses’ knowledge and self-efficacy**

|  | **Intervention clusters n (%)** | | | | | | **Control clusters n (%)** | | | | | |
| --- | --- | --- | --- | --- | --- | --- | --- | --- | --- | --- | --- | --- |
|  | Strongly disagree | Disagree | Neither agree or disagree | Agree | Strongly agree | Don’t know | Strongly disagree | Disagree | Neither agree or disagree | Agree | Strongly agree | Don’t  know |
| I can confidently provide care to pregnant women | - | - | - | 1 (4.3) | 22 (95.7) | - | - | - | - | 3 (9.4) | 29 (90.6) | - |
| I can confidently provide basic knowledge about pregnancy to the pregnant women I care for | - | - | - | 1 (4.3) | 22 (95.7) | - | - | - | - | 3 (9.4) | 29 (90.6) | -- |
| I can confidently recognize pregnancy related complications | - | - | - | 1 (4.3) | 22 (95.7) | - | - | - | - | 2 (6.3) | 30 (93.7) | - |
| I can confidently recognize danger signs / symptoms related to pre-eclampsia | - | - | 1 (4.3) | - | 22 (95.7) | - | - | - | - | 1 (3) | 31 (97) | - |
| I can confidently recognize danger signs / symptoms related to eclampsia | - | - | - | 1 (4.3) | 22 (95.7) | - | - | - | - | 1 (3) | 31 (97) | - |
| I am confident that I can recognize signs and symptoms of labor | - | - | - | 3 (13) | 20 (87) | - | - | - | - | - | 32 (100) | - |
| I am confident that I can accurately check blood pressure | - | - | - | - | 23 (100) | - | - | - | - | 1 (3) | 31 (97) | - |
| The training I received from the CLIP team provided the skills I needed to take care of pregnant women in the CLIP Trial | - | - | - | 3 (13) | 20 (87) | - | - | - | - | 2 (6.3) | 30 (93.7) | - |
| I could count on the support of the supervision to solve problems encountered in identifying pregnant women | - | - | - | 4 (17.4) | 19 (82.6) | - | - | - | - | 4 (12.5) | 28 (87.5) | - |
| Women/families in my community seek my advice on pregnancy and maternal health | - | - | - | 4 (17.4) | 19 (82.6) | - | - | - | - | 6 (18.8) | 26 (81.2) | - |
| I feel comfortable accompanying a pregnant woman who needs medical attention to the hospital | - | - | 1 (4.3) | 3 (13) | 19 (82.6) | - | - | - | 1 (3) | 31 (97) | - | - |
| Hospital doctor respect my judgment when I identify that a pregnant woman needs my care | - | 1 (4.3) | - | 3 (13) | 19 (82.6) | - | - | - | - | - | 32 (100) | - |
| If a woman needs of clinical care, your family takes seriously the advice | - | 1 (4.3) | - | 2 (8.7) | 20 (87) | - | - | - | - | 7 (21.9) | 25 (78.5) | - |
| Families trust the health care I provide to pregnant women in my community | - | - | 1 (4.3) | 3 (13) | 19 (82.6) | - | - | - | - |  | 32 (100) | - |
| I am good to advise and convince women that need attention | - | - | - | 6 (26) | 17 (74) | - | - | - | - | 4 (12.5) | 28 (87.5) | - |
| Women in my community trust in my abilities | - | 1 (4.3) | - | 5 (21.7) | 17 (74) | - | - | - | - | 2 (6.3) | 30 (93.7) | - |
| Doctors in my facility view my role as a nurse as important | - | - | 1 (4.3) | 1 (4.3) | 21 (91.4) | - | - | - | - | - | 31 (97) | 1 (3) |
| I feel comfortable working with the CHW’s in my community | 1 (4.3) | - | 1 (4.3) | 3 (13) | 17 (74) | 1 (4.3) | - | - | - | 1 (3.1) | 12 (37.5) | 19 (59.4) |
| I trust the assistance provided by CHW’s in my community | 1 (4.3) | - | 2 (8.7) | 3 (13) | 16 (70) | 1 (4.3) | - | - | - | 6 (18.7) | 7 (21.9) | 19 (59.4) |
| CHW’s activity in my community helps women to go to ANC early | 1 (4.3) | 1 (4.3) | - | 3 (13) | 18 (78) | - | - | - | - | 1 (3.1) | 12 (37.5) | 19 (59.4) |
| CHW’s activity help me to improve the identification and referral of women with obstetric emergencies | 1 (4.3) | 1 (4.3) | 1 (4.3) | 2 (8.7) | 18 (78) | - | - | - | - | 2 (6.3) | 11 (34.4) | 19 (59.4) |
| CHW’s activity in my community helps to refer pregnant women more quickly | 1 (4.3) | - | 1 (4.3) | 2 (8.7) | 19 (82.6) | - | - | - | - | 2 (6.3) | 11 (34.4) | 19 (59.4) |

**Table S3. CHW’s knowledge and self-efficacy**

|  | **Intervention clusters n (%)** | | | | | | **Control clusters n (%)** | | | | | |
| --- | --- | --- | --- | --- | --- | --- | --- | --- | --- | --- | --- | --- |
|  | Strongly disagree | Disagree | Neither agree or disagree | Agree | Strongly agree | Don’t know | Strongly disagree | Disagree | Neither agree or disagree | Agree | Strongly agree | Don’t  know |
| I can confidently provide care to pregnant women | - | - | - | 18 (41) | 26 (59) | - | 4 (5.4) | 4 (5.4) | 1 (1.4) | 24 (32.4) | 41 (55.4) | - |
| I can confidently provide basic knowledge about pregnancy to the pregnant women I care for | - | - | - | 17 (38.6) | 27 (61.4) | - | 3 (4.1) | 3 (4.1) | 2 (2.7) | 24 (32.4) | 42 (56.8) | - |
| I can confidently recognize pregnancy related complications | - | - | - | 17 (38.6) | 27 (61.4) | - | 2 (2.7) | 4 (5.4) | 1 (1.4) | 24 (32.4 | 43 (58.1) | - |
| I can confidently recognize danger signs / symptoms related to pre-eclampsia | - | - | - | 15 (34) | 29 (66) | - | 39 (52.7) | 20 (27) | 1 (1.4) | 10 (13.5) | 4 (5.4) | - |
| I can confidently recognize danger signs / symptoms related to eclampsia | 1 (2.3) | - | - | 16 (36.4) | 27 (61.3) | - | 40 (54.1) | 20 (27) | - | 12 (16.2) | 2 (2.7) | - |
| I am confident that I can recognize signs and symptoms of labor | 1 (2.3) | - | - | 16 (36.4) | 27 (61.3) | - | 2 (2.7) | 5 (6.7) | 2 (2.7) | 21 (28.4) | 44 (59.5) | - |
| I am confident that I can accurately check blood pressure | - | - | - | 14 (32) | 30 (68) | - | 23 (31.1) | 21(28.4) | 3 (4.1) | 14 (18.9) | 13 (17.6) | - |
| The training I received from the CLIP team provided the skills I needed to take care of pregnant women in the CLIP Trial | - | - | - | 13 (29.6) | 30 (68) | 1 (2.3) | NA | NA | NA | NA | NA | NA |
| I could count on the support of the supervision to solve problems encountered in identifying pregnant women | - | 1 (2.3) | - | 15 (34) | 28 (63.6) | - | 2 (2.7) | 3 (4.1) | - | 25 (33.8) | 44 (59.5) | - |
| I can confidently administer injectable vaccines to mothers and child | 4 (9.1) | 3 (6.8) | - | 14 (31.8) | 23 (52.3) | - | 20 (27) | 18 (24.3) | 1 (1.4) | 20 (27) | 15 (20.3) | - |
| I can confidently administer intramuscular injections to pregnant women at risk of seizures | - | 1 (2.3) | - | 13 (29.6) | 30 (68.1) | - | 26 (35.1) | 32 (43.2) | 2 (2.7) | 8 (10.8) | 6 (8.1) | - |
| Women/families in my community seek my advice on pregnancy and maternal health | - | - | - | 17 (38.6) | 27 (61.3) | - | 1 (1.4) | 3 (4.1) | - | 26 (35.1) | 43 (58.1) | 1 (1.4) |
| I feel comfortable accompanying a pregnant woman who needs medical attention to the hospital | - | - | - | 14 (31.8) | 30 (68.1) | - | - | - | 1 (1.4) | 23 (31.1) | 50 (67.6) | - |
| Hospital nurse respect my judgment when I identify that a pregnant woman needs my care | - | - | 1 (2.3) | 16 (36.4) | 27 (61.3) | - | - | 3 (4.1) | 3 (4.1) | 25 (33.8) | 43 (58) | - |
| If a woman needs of clinical care, your family takes seriously the advice | - | - | - | 14 (31.8) | 30 (68.1) | - | - | - | 2 (2.7) | 28 (37.8) | 44 (59.5) | - |
| Families trust the health care I provide to pregnant women in my community | - | - | - | 16 (36.4) | 28 (63.6) | - | - | - | 1 (1.4) | 28 (37.8) | 45 (60.8) | - |
| I am good to advise and convince women that need attention | - | - | 1 (2.3) | 13 (29.6) | 28 (63.6) | 2 (4.5) | - | 1 (1.4) | - | 25 (33.8) | 48 (64.9) | - |
| Women in my community trust in my abilities | - | - | - | 12 (27.3) | 31 (70.5) | 1 (2.3) | - | - | 2 (2.7) | 23 (31.1) | 49 (66.2) | - |
| Doctors in my facility view my role as a nurse as important | - | - | - | 14 (31.8) | 30 (68.1) | - | 1 (1.4) | 2 (2.7) | 1 (1.4) | 23 (31.1) | 47 (63.5) | - |

**Table S4. CHW responses related to use of the POM app**

|  | **Intervention clusters n (%)** | | | | | |
| --- | --- | --- | --- | --- | --- | --- |
|  | Strongly disagree | Disagree | Neither agree or disagree | Agree | Strongly agree | Don’t know |
| The POM app was easy to use | 1 (2.3) | 8 (18.2) | - | 15 (34.1) | 20 (45.5) | - |
| I was effectively trained to use the POM mobile application in the CLIP Trial | - | - | - | 18 (40.9) | 25 (56.8) | 1 (2.3) |
| The training I received from the CLIP team provided the skills I needed to care for pregnant women in the CLIP Trial | - | - | 1 (2.3) | 17 (38.6) | 26 (59.1) | - |
| I could rely on the support of the CLIP team to solve problems encountered when using the POM app | 2 (4.5) | 2 (4.5) | - | 18 (41) | 22 (50) | - |
| The pregnant women I cared for always reacted positively to my use of the POM app | 1 (2.3) | 1 (2.3) | - | 17 (38.6) | 24 (54.5) | 1 (2.3) |
| I felt good about using the POM app | - | - | - | 13 (29.6) | 31 (70.4) | - |
| I often had trouble completing study visits using the POM app | 25 (56.8) | 6 (13.6) | - | 11 (25) | 2 (4.5) | - |
| The pregnant women I cared for generally expressed negative views about using the POM application during my care | 28 (68.6) | 13 (29.6) | 1 (2.3) | 1 (2.3) | 1 (2.3) | - |
| My supervision actively supported my use of the POM app | - | 2 (4.5) | - | 17 (38.6) | 24 (54.5) | 1 (2.3) |
| The pictograms in the application were useful in helping me counsel women | - | - | - | 20 (45.5) | 24 (54.5) | - |
| The pictograms adequately conveyed the symptoms they were supposed to represent | - | - | - | 18 (41) | 26 (59) | - |
| The refresher trainings helped me overcome obstacles | - | - | 1 (2.3) | 18 (41) | 25 (56.8) | - |
| The POM app was unnecessary to advise women about pregnancy complications | 24 (54.5) | 6 (13.6) | - | 5 (11.4) | 8 (18.2) | 1 (2.3) |
| Women took my counselling more seriously because of the POM app | 4 (9.1) | 6 (13.6) | 1 (2.3) | 16 (36.4) | 17 (38.6) | - |
| I would recommend use of the POM app to other health workers | 2 (4.5) | 1 (2.3) | - | 16 (36.4) | 25 (56.8) | - |
| If offered, I would choose to continue using the POM app to care for pregnant women | - | - | - | 12 (27.3) | 32 (72.7) | - |
